# Investigating the Association between Outdoor Environment and Outdoor Activities for Seniors Living in Old Residential Communities

**DOI:** 10.3390/ijerph18147500

**Published:** 2021-07-14

**Authors:** Shiwang Yu, Na Guo, Caimiao Zheng, Yu Song, Jianli Hao

**Affiliations:** 1 School of Civil Engineering, Sanjiang University, Nanjing 210012, China; Sherwood.s.w.yu@gmail.com (S.Y.); 12017082031@stu.sju.edu.cn (C.Z.); 2 College of Economic and Management, Nanjing Institute of Industry Technology, Nanjing 210023, China; Guon@niit.edu.cn; 3 XIPU Institution, Xi’an Jiaotong-Liverpool University, Suzhou 215123, China; yu.song@xjtlu.edu.cn; 4 Department of Civil Engineering, Xi’an Jiaotong-Liverpool University, Suzhou 215123, China

**Keywords:** outdoor environment, daily outdoor activities, old residential communities, seniors

## Abstract

Many seniors live in old residential communities (ORCs) with low-quality outdoor environment (OE), which hinders the residents’ outdoor daily activities (ODAs). This paper empirically investigates the association of OE on ODAs for seniors living in ORCs. A questionnaire was designed and distributed in six central districts of Nanjing city. A total of 258 questionnaires was finally collected, of which 60.08%, 29.46%, 9.69%, and 0.78% respondents were scattered into four age groups (61–69, 70–79, 80–89, and ≥90), respectively. Based on reliability analysis, correlation analysis, and regression analysis, the results show that: (1) social activities are mainly associated with noise; (2) leisure activities are significantly associated with road accessibility, slip-resistance measures, greenery, and staff; (3) utilitarian-type activities are significantly associated with stairway accessibility, slip-resistance measures, greenery, and seating; (4) there is a significant association between nature-exposure activities and layout, greenery, and poor air quality. The findings could guide Chinese officials when renewing ORCs by addressing the most important outdoor environmental factors associated with ODAs.

## 1. Introduction

China has become an aging society, with 264.02 million seniors aged 60 years and over in 2020 [1], and it is estimated that the number of seniors will increase to 500 million by 2050 [2]. Such a severe aging population can cause problems in society due to a weakened supportive environment in terms of physical, psychological, financial, medical, and social needs. Evidence shows that an aging population increase is followed by a decrease in the birth rate, giving rise to even more serious social problems [3]. The number of seniors with inadequate support in China has significantly increased in recent years due to government policies resulting in couples having fewer children and people being too busy to practically care for their elderly parents [4,5].

Although there is a trend of urban renewal and consequential residential resettlements in China, most seniors prefer to stay in the home where they have lived for an extended period and where, for financial and psychological reasons, they can live with dignity [6,7]. However, most of their apartments are located in old residential communities (ORCs) where the outdoor environment (OE) barely exists and cannot adequately support them as they grow older. According to the General Office of the State Council of the People’s Republic of China, ORCs refer to residential communities that were built in an earlier age, are poorly maintained, have no management, lack municipal and community service facilities, and where residents have a strong desire for renewal [8]. Compared to younger residents who go out to work each day, seniors spend more time living in ORCs. The outdoor daily activities (ODAs) of seniors in ORCs are significantly associated with the outdoor environment in ORCs [9]. However, unlike the institutions for the older adults including nursing homes and home for the aged, which are specifically designed for the older adults, the quality of the outdoor environment in these OCRs is quite poor and unfriendly to the residents, especially the older ones. Scholars in China have reported that poor the quality of facilities in ORCs are troubling for many older residents [10]. To meet the desire of the people for a better-quality life, the State Council issued “Guiding Opinions of the General Office of the State Council on Comprehensively Promoting the Renewal of Old Residential Communities in Cities and Towns” to improve the facilities of ORCs. This policy is especially beneficial for the older adults, since they make up the majority of residents living in ORCs [11].

There have been many studies dealing with the association of the living environment in ORCs on seniors’ quality of life from city planning and psychological aspects [12,13,14], and OE factors for older adults (accessibility, the physical environment, and supporting facilities) have been widely studied in developed regions [15,16,17,18]. However, unlike communities in other countries, most of the ORCs in China are gated communities, and their OE, which is surrounded by a guarded gate and wall or fence, is jointly owned by all the homeowners [19]. Although residents living in these gated communities enjoy the benefits of being away from the disturbance of public transportation and strangers, they have to pay for the cost of the management and maintenance of the OE. One of the significant reasons for the poor quality of OE in ORCs is the lack of effective management and maintenance due to a lack of financial resources. Furthermore, due to different culture, customs, and lifestyle, the common outdoor environment factors between China and other countries are usually not consistent, and therefore, findings from other types of residential communities cannot be applied to OCRs in China. Moreover, the existing ORC-related research has mostly focused on a sponge-style community approach, evaluation methods, and public participation [20,21,22,23]. There is a scarcity of studies focusing on the association between OE in ORCs and seniors’ outdoor daily activities. Given the large size and fast growth of the older population and plenty of ORCs in China, there is a need to investigate ways to improve the OE of ORCs in order to encourage more ODAs for older adults.

## 2. Literature Review

### 2.1. Outdoor Environment Factors

Previous studies have found many outdoor environment factors associated with the daily life of seniors. These factors are classified as either accessibility, the physical environment, or supporting facilities.

The accessibility of a residential community refers to the layout and elevation design of the buildings and outdoor environment to provide an accessible environment of freedom, communication, and community [18,24,25]. Demura et al. (2012) found that stairway accessibility could help seniors avoid tripping or slipping when walking up and down [26]. A reasonable residential layout leads to an optimized solution across several conflicting criteria by taking care of most residents’ interests and providing a convenient and safe environment for their daily activities, while a poor layout can cause inconvenience and trouble to the residents [27,28,29]. Inaccessible roads/paths with uneven surfaces, curbs, and potholes will pose a hazard to residents, especially seniors [30,31]. All the factors mentioned regarding accessibility, significantly contribute to a senior’s decision to pursue outdoor daily activities.

The outdoor physical environment, which includes lighting, noise, and air quality, is inevitably associated with the physical comfort of outdoor spaces [32,33]. Poor lighting along roads/paths could give rise to difficulties and even be hazardous for seniors [34]. Noise, which can be classified as unwanted sound, often interrupts on-going activities and induces annoyance [35,36]. Air quality refers to the physical, chemical, and biological characteristics of air, which is important for people’s health and activities [37]. Older adults usually experience the aforementioned outdoor physical environment factors when conducting their outdoor activities.

Supporting facilities in ORCs provide a convenient and comfortable environment for its senior residents, the most common of which are greenery, handrails, fitness equipment, security, and seating [18]. Senior residents can be quickly drawn to the greeneries to rest, socialize, and take exercise [38,39]. Since seniors are more prone to fall without the help of handrails due to their decreased physical ability [40], certified handrails could be installed to provide a supportive environment for seniors [41]. A lack of security measures can trigger fear or worry and make seniors hesitant to go outside [42]. Cleaning work provides a tidy environment by removing trash from public areas where residents usually walk or stay around [43]. Fitness equipment provides an opportunity for people to take part in leisure activities, while a lack of it may lead to less physical activities for seniors [44,45]. Availability of seating is a significant concern for older adults when they think about going out [46,47]. It is very important for facilities management staff to behave in a friendly manner, respond to the residents’ concerns quickly, and try to meet their needs [48]. Failing to do so often leads to residents feeling dissatisfied and even to complaints [49]. All of the aforementioned supporting facilities play a significant part in promoting residents’ satisfaction with their daily lives.

### 2.2. Outdoor Daily Activities

Outdoor daily activities are necessary for seniors’ physical and social needs [50]. There are broadly four types of ODAs for seniors: (1) social activities; (2) leisure activities; (3) activities of daily living, and (4) nature-exposure activities [51,52,53,54,55]. These ODAs are essential for seniors’ quality of life.

Social activities refer to the activities focusing on social contact with other people in different ways, including meeting with family members and gathering with friends [56]. Chatting with neighbors and social interaction with friends in outdoor spaces are everyday social activities for seniors [57,58]. Participating in social activities is an efficient way to maintain and improve social relations with health benefits [59]. Frequent participation in social activities has been shown to contribute to a decreased risk of dementia [60].

Leisure activities are very important to the physical and mental wellbeing of seniors [61,62]. There are many kinds of leisure activities, including tai chi, Mahjong, cards, walking, gardening, shopping, and vigorous exercise [63]. Regular participation in leisure activities can contribute to many health and social benefits, such as happiness, life satisfaction, and even a lower risk of dementia [64,65]. Taking part in leisure activities provides seniors with opportunities to participate in society and to lead a fuller life.

Seniors usually spend a lot of time at home performing basic activities of daily living and need to go outside to participate in utilitarian-type activities (UTA), such as using public transportation, going to the bank, and shopping for groceries [52,66]. However, seniors’ utilitarian-type activities can be stymied by environmental barriers, such as poor road conditions or long distances [42,67,68]. A supportive environment is necessary for seniors to perform utilitarian-type activities.

Nature-exposure activities (NEAs) refer to the activities closely connected with the natural environment, including sunshine, water, greenery, and animals. Seniors can take part in nature-exposure activities in many ways, such as exposure to the sun, enjoying natural elements (garden/landscaped areas), and listening to the sound of birds and running water [69,70]. Evidence shows that NEAs are conducive to improved happiness, satisfaction, and wellbeing, while also helping to control anger, stress, and blood pressure [71,72,73,74].

## 3. Methodology

### 3.1. Conceptual Model

**Figure 1:**
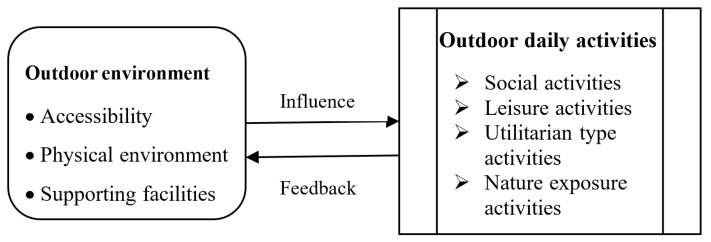
Conceptual model of OE and ODAs for seniors in ORCs.

Relevant literature reveals four types of outdoor daily activities of older adults associated with accessibility, physical environment, and supporting facilities in different living environments. A conceptual model is proposed to explain the association between outdoor environment and ODAs (social activities, leisure activities, activities of daily living, and nature-exposure activities) for seniors. As such, the hypothesis for this research is that there is a significant association between outdoor environment factors of OCRs and the outdoor daily activities (ODAs) of seniors. A conceptual model is shown in Figure 1.

### 3.2. Survey Design

A questionnaire was created for investigating the association between OE on ODAs for seniors, which included: (1) demographic characteristics, (2) outdoor daily activities components, and (3) outdoor environment components.

To collect the background information of respondents, the first part of the questionnaire sought demographic characteristics of respondents, namely gender, age, education level, and period of residence in their residential community. In the second section, four types of ODAs, namely social activities, leisure activities, outdoor activities of daily living, and nature-exposure activities were adopted based on previous studies [51,52,53,54,75]. The third part of the questionnaire included fourteen OE factors based on a review of relevant literature, China’s “Property Management Regulations” and “Evaluation Standards for Service Quality of Provincial Demonstration Facilities Management Projects in Jiangsu Province” [18,76,77,78,79]. All the questions were slightly adjusted to fit the actual situation of the ODAs and outdoor environment in ORCs in China (refer to Table 1). More details of questionnaire development can be found in supplementary file S1. A total of 16 questions regarding ODAs and 53 questions with respect to outdoor environment were included in the questionnaire. A five-point Likert scale was adopted to measure the respondents’ perception on the items of the questionnaire where 1 indicated extremely infrequently or strongly disagree and 5 indicated extremely frequently or strongly agree. The score for the level of agreement with each OE component and ODAs domain was calculated by taking the average of the ratings of relevant items.

### 3.3. Sample

To select a representative sample, the stratified random sampling method was employed in this research [61]. Sixteen ORCs from six districts in the city of Nanjing were finally selected with the following choice criteria: (1) completion time; (2) community size; (3) number of the residents; (4) location. Senior respondents in these OCRs were selected with the following criteria: (1) age 60 years and above); (2) minimum one year of residency; (3) state of health. State of health was adopted as a threshold so that only those seniors whose health allowed them to stay in the outside environment in ORCs were invited to participate in this study. Residents who rarely went outside because of serious illness, such as dementia or physical disability, were not considered in this study. The survey was conducted between March and July 2019.

Many seniors may have difficulties in filling out the questionnaire due to vision problems and/or poor education. To improve the response rate and ensure each respondent fully understood the meaning of each question, where considered necessary, questionnaires were distributed personally to respondents by well-trained investigators who explained every question in the questionnaire so that the respondents could fully understand and offer the most appropriate response.

From the 600 distributed questionnaires, 258 valid ones were returned giving a response rate of 43%. Pre-analysis screening was firstly conducted to check the missing data. No patterns were found in the missing data, and all the missing data were dispersed randomly. Mean substitution was employed to deal with missing data during data analysis, which has been adopted in some studies (e.g., [92]). Among the four age groups of respondents, the first age group of 60- to 69-year-olds accounted for the biggest proportion at 60.08%, which was followed by the second age group of 70- to 79-year-olds at 29.46%. The third age group of 80- to 89-year-olds and the fourth age group of 90 plus were 9.69% and 0.78%, respectively. Detailed information of the respondents is listed in Table 2.

Bonferroni adjustment was made to control Type I error. The questionnaire comprised 69 questions, and the adjusted *p*-value was 0.0007 (0.05/69). According to Levene’s test, there was no difference in the variables for the respondents in ORCs, indicating that the variable data had variability and homogeneity (refer to the supplementary file S2). Based on this, the variable data were combined in this study.

## 4. Results

### 4.1. Reliability Test of the OE Factors and the ODAs Domains

Reliability analysis was employed during data analysis to check the extent to which items in the questionnaire were related to each other and to provide an overall index of the internal consistency of the scale. Cronbach’s alpha was adopted to measure the reliability of each factor, and the value of 0.6 was taken as the threshold [93,94]. After confirming the reliability of the construct, all the item ratings were summed up to predict the 14 OE factors: stairway accessibility (F1), road accessibility (F2), layout (F3), slip-resistance measures (F4), noise (F5), poor air quality (F6), lighting (F7), greenery (F8), handrail (F9), security (F10), cleaning (F11), fitness equipment (F12), seating (F13), and staff (F14) (see Table 3). Four types of ODAs, social activities (ODA1), leisure activities (ODA2), utilitarian-type activities (ODA3), and nature-exposure activities (ODA4), were also identified from the reliability analysis (see Table 4).

### 4.2. Correlation Analysis of OE and ODAs

The results of the correlation analysis show that there are significant associations between seniors’ outdoor daily activities and OE factors, as shown in Table 5. Social activities have significant associations with five OE factors: layout (F3: 0.246), slip-resistance measures (F4: 0.245), noise (F5: −0.417), poor air quality (F6: 0.292), and cleaning (F11: 0.222). Leisure activities were revealed to significantly correlate with three OE factors: road accessibility (F2: 0.245), slip-resistance measures (F4: 0.272), and staff (F14: 0.293). Activities of daily living were positively significantly correlated with stairway accessibility (F1: 0.336), while negatively significantly correlated with poor air quality (F8: −0.269), cleaning (F11: −0.317), fitness equipment (F12: −0.427), and seating (F13: −0.404). Nature-exposure activities have significant association with stairway accessibility (F1: 0.223), road accessibility (F2: 0.292), layout (F3: 0.365), greenery (F5: −0.422), poor air quality (F6: −0.287), cleaning (F11: 0.244), fitness equipment (F12: 0.235), and seating (F13: 0.236).

### 4.3. Multiple Regression Analysis

Four separate multiple regressions were conducted using a stepwise method to test the predictive power and relative contribution of the OE factors for each ODA [82,95]. Demographic factors, such as age gender and period of residence, were not included in the final models, since this research focused on the OE factors (see Table 6).

Social activities (ODA1) were positively associated with slip-resistance measures (F4), while negatively associated with noise (F5), explaining 22.2% of the variance in Model 1. In Model 2, leisure activities (ODA2) were positively associated with road accessibility (F2), slip-resistance measures (F4), greenery (F8), and staff (F14), explaining 22.3% of the variance. Model 3 showed that activities of daily living (ODA3) were negatively induced by greenery (F8) and seating (F13), while positively associated with stairway accessibility (F1) and slip-resistance measures (F4), accounting for 35.2% of the variance. Model 4 revealed that nature-exposure activities were negatively associated with poor air quality (F6) and positively associated with layout (F3) and poor air quality (F6), explaining 16.4% of the variance.

## 5. Discussion

The results reveal in both correlation and regression analysis that four factors of accessibility, two factors of physical environment, and three factors of supporting facilities are associated with ODAs.

### 5.1. Factors Significantely Associated with Social Activities

Slip-resistance measures are revealed to be positively associated with social activities. Due to declining physical health, seniors are often worried about their personal safety. The slippery surface of the road can easily lead to younger adults falling, let alone frail seniors [96]. Seniors may hesitate to go out if they know there are many slippery surfaces in the area or may be encouraged to go outside if slip-resistant measures, such as anti-slip mats and non-slip tiles, are in place.

Hearing is one of the first senses to be affected by age, which starts to wane by the age of 40 [97]. Even for the seniors who have normal hearing, too high noise in ORCs, including life noise, transportation noise, construction noise, and traffic noise, makes it hard for seniors to clearly hear conversation-level voices. In addition to communication difficulties, too much noise will also induce negative feelings, such as depression and anxiety [98]. Hence, the social activities of seniors will often be hindered or even obstructed by severe noise.

### 5.2. Factors Positively Associated with Leisure Activities

Road accessibility is another OE factor associated with leisure activities in the outdoor living environment of ORCs. Seniors often need to walk along roads/paths to reach outdoor leisure activities or to simply take a stroll in ORCs. However, there are often many barriers including potholes and curbs due to lack of proper and timely maintenance. Seniors’ physical functions decline with age and often find small obstacles or uneven surfaces to be hazardous [99,100]; uneven roads/paths are often reported by older adults [101]. Safety issues surrounding poorly maintained roads/paths will discourage seniors from walking for leisure or for brisk exercise. On the other hand, well maintained roads/paths can create safe and comfortable walking routes for seniors and encourage them to go outside for leisure activities in ORCs [77].

Slip-resistance measures, discussed above in relation to its association with seniors via social activities, were also revealed to be positively and significantly associated with leisure activities. Well-maintained roads/paths can encourage seniors to walk outside to take exercise, while poorly maintained roads/paths and slippery surfaces would make them hesitate to go outside [42]. Greenery is positively associated with leisure activities, which was shown by the regression analysis. Within an urban environment, greenery provides favorable living conditions [102], and seniors prefer to stay in their community and walk around green spaces during the time they exercise outside [103]. As such, it is suggested that local governments and councils focus on maintaining and increasing greenery-filled public areas that are convenient to walk in and are within easy walking distance of each residential building [104].

### 5.3. Factors Associated with Utilitarian-Type Activities

Elevators were not installed in most of the residential buildings of the OCRs in China [105]. Residents in ORCs have to go in and out of the building through the entrance and stairway in all the surveyed ORCs. Unlike younger adults, stairways present seniors with many difficulties; they may, for example, easily trip over obstacles left on the stairway and fall. Falls are the leading cause of both fatal and non-fatal injuries among seniors [106]. Hence, stairway accessibility is an important factor for seniors, which is confirmed by this study. Barriers in the building will decrease seniors’ intention to take outdoor utilitarian-type activities (UTA), including going to the market and post office. They will tend to stay at home and not go out to buy necessities, take a walk, or visit family members and friends. This inactivity can easily induce depression, stress, and anxiety [107].

Slip-resistance measures are revealed to be positively associated with outdoor UTA. Since seniors often need to walk on the road to go outside of the ORCs, any slippery surface on the road could be a serious hazard for them. Hence, seniors may have a fear of going outdoors due to the dangers of slippery surfaces [42,108].

Seating along the road and in recreational areas is an important facility for people to take a rest, and seat availability is a major concern for seniors [109]. However, it is interesting that seating was found to be negatively associated with UTA. A reasonable explanation for this finding would be that seniors who plan outdoor UTA might be tempted to take a rest on a comfortable seat and start chatting with neighbors and friends instead of continuing with their UTA; after all, most seniors have ample time to deal with personal issues after retirement. However, if the seating is not well maintained, they may only take a rest for a short while and then continue with their UTA.

Greenery was found to negatively promote outdoor UTA. As discussed above, favorable greenery has many physical and psychological benefits [110]. When going out to take UTA such as playing Mahjong and square dancing, seniors might be attracted by the greenery in the neighborhood and, thus, change their UTA plan.

### 5.4. Factors Significantly Associated with Nature-Exposure Activities

Good greenery can provide a collection of green bushes, leaves, and flowers that can increase an individual’s perspective of their living environment, while unkempt greenery could evoke a negative response from seniors [38,111]. Hence, seniors would like to undertake activities that expose them to nature, including enjoying the benefits of good scenery and sunshine [71,112]. Conversely, seniors will not be willing to go outside for nature-exposure activities if outdoor greenery is poor and insufficient.

Poor air quality was revealed in this study to deter nature-exposure activities. Fresh air can encourage seniors to go outside, since the indoor quality of air is usually poor due to inadequate ventilation and domestic odors. Seniors can also receive physical benefits from fresh air. However, if the outside air quality is poor (due to domestic trash, for example), seniors may choose not to partake in nature-exposure activities.

Layout can also be positively and significantly associated with seniors’ nature-exposure activities. A poor layout is regarded as posing potential problems and hazards for seniors [113,114]. Seniors would hesitate to take nature-exposure activities when ORCs have a poor layout, such as greenery too far from seniors’ residences, insufficient public spaces, or too many vehicles in the area. However, a favorable layout with safe, easily accessible public areas can create a satisfying living environment for seniors.

## 6. Limitations of the Study

Although several important findings emerged from this research, the following limitations should be noted. Firstly, the questionnaire survey’s relatively small sample size and self-reporting subjective measurement for environmental factors may limit the generalizability of the results and call into question the potential common method variance and the validity of data. However, the following factors decrease such possibility: (1) all scales for measurement of OE factors and ODAs were adopted based on an extensive literature review; (2) the senior respondents in this study were intentionally selected with different backgrounds, such as age, gender, and health status; (3) all respondents selected in this research came from different ORCs and different old districts in Nanjing city; (4) all the senior respondents had lived in their ORCs for at least one year and, thus, had a direct experience of the OE in their community; (5) all the factors adopted in this study were statistically tested as reliable (greater than 0.6). The research team is, therefore, confident that the results of this research have not been biased by different responses to the measured variables and can be used as baseline information for further larger-scale studies with regards to the association between OE factors in ORCs and the quality of life of senior residents. It can also be treated as a reliable reference for research with different types of residential communities, such as settlement building communities, new-build residential communities, and public rental housing.

Secondly, the demographic questions in the questionnaire acquired age, gender, period of residence, educational level, marital status, and faith. The financial and living arrangement of the older adults were not included for the following reasons: (1) this research mainly aims to investigate the important OE factors influencing the ODAs of older adults and provide suggestions to improve the OE for older adults in ORCs during the process of renewal of ORCs in cities and towns in China; therefore, the OE factors were more focused on; (2) although financial and living arrangements are essential to predict older adults’ health behaviors, they have little relation with the outdoor environment, which is a public area rather than a private place; (3) older adults have to stay in or pass through the OE in ORCs to take part in ODAs, regardless of their financial and living arrangement. However, these two important factors should be considered in future research on the association between indoor environment and the quality of life or safety of older adults.

Thirdly, mean substitution (MS) was adopted in this study to deal with missing data. However, since the MS method has been criticized in some studies (e.g., [115]), the effectiveness of the data analysis in this study is questionable. It is, therefore, suggested to apply multiple imputation and the full information maximum likelihood method in further studies [115].

Fourthly, Bonferroni adjustments were not used during the regression analysis, and the alpha criterion was set at 0.05, which might increase the type-I error rate. However, adopting Bonferroni adjustments may lead to a type-II error [116]. This method is too conservative [117], especially when there is a large number of comparisons to be made.

## 7. Conclusions and Implications

This research revealed some important findings: there is a significant association between social activities and slip-resistance measures as well as noise in ORCs; leisure activities can be associated with road/path accessibility, slip-resistance measures, greenery, and staff; utilitarian-type activities are associated with stairway accessibility, slip-resistance measures, greenery, and seating; nature-exposure activities are associated with layout, air quality, and greenery.

Based on the findings of this research, practical measures can be implemented to provide a quality outdoor environment for seniors in the process of renewing ORCs in China. Given the association between road accessibility and leisure activities, asphalt road surfaces are advised instead of concrete and brick, since it has the advantages of good mechanical strength, a smooth surface, and needing relatively little maintenance [118]. Ramps should be built along pathways instead of steps. Due to the association between slip-resistance measures and three types of ODAs, the following strategies can be adopted to encourage the ODAs of seniors: scattering straw or salt on wet or snowy days and cleaning up immediately after the rain or when the snow stops. However, the health situation and walking ability of the older respondents may influence the final model regarding ODAs and OE factors. It is suggested that future studies explore the association between ODAs and OE factors under different types of health and walking abilities of older residents.

## Figures and Tables

**Table 1:** Factors adopted in this study and their sources.

Category	Factors	Source
Outdoor daily activities	ODAS1—Social activities	Wen et al., 2010 [75]
	ODAS2—Leisure activities	Wen et al., 2010 [75]
	ODAS3—Utilitarian-type activities	Mahmood et al., 2012, Zhao et al., 2017 [66,80]
	ODAS4—Nature-exposure activities	Wen et al., 2010 [75]
Accessibility	F1—Stairway accessibility	Leung et al., 2016, 2017 [81,82,83]
	F2—Road/path accessibility	Brownson et al., 2004, Laura N. Gitlin, 2001 [84,85]
	F3—Layout	Sun, 2012 [86]
	F4—Slip-resistance measures	Leung et al., 2016 [81]
Physical environment	F5—Noise	Ma et al., 2018 [87]
	F6—Poor air quality	Sun, 2016 [88]
	F7—Lighting	Leung et al., 2019 [89]
Supporting facilities	F8—Greenery	DHURDJP, 2019 [78]
	F9—Handrail	Leung et al., 2019 [89]
	F10—Security	DHURDJP, 2019 [78]
	F11—Cleaning	DHURDJP, 2019 [78]
	F12—Fitness equipment	Tang, 2018 [90]
	F13—Seating	Tang, 2014 [91]
	F14—Staff	DHURDJP, 2019 [78]

**Table 2:** Detailed information of respondents.

Items	Details	Amount	Proportion (%)
Age	61–69	155	60.08%
	70–79	76	29.46%
	80–89	25	9.69%
	≥90	2	0.78%
Gender	Male	127	49.22%
	Female	131	50.78%
Period of residence	1–5 y	42	16.28%
	6–10 y	33	12.79%
	11–15 y	108	41.86%
	16–20 y	26	10.08%
	>20 y	49	18.99%
Education level *	Primary school	120	46.88%
	Junior middle school	73	28.52%
	Senior middle school	56	21.88%
	Bachelor and above	7	2.73%
Marital status	Never married	3	1.16%
	Married	198	76.74%
	Widowed	57	22.09%
Faith	Christianity	6	2.33%
	Buddhism	21	8.14%
	Marxism	18	6.98%
	No faith	208	80.62%
	Others	5	1.94%

Note: *—2 were missing.

**Table 3:** Reliability analysis of OE factors in ORCs.

Factors	Items	Details	Valid Observations	Alpha (α)
F1—Stairway accessibility	1.	Seniors using wheelchair or walking stick can go through	252	0.695
	2.	No stuff stacked along the stairway		
	3.	Tiles used in stairway was non-slip		
	4.	Ramps were set at steps		
F2—Road/path accessibility	5.	Roads/paths are easy to walk on	258	0.809
	6.	Manhole covers are kept well		
	7.	No hollow and holes on the pavement		
	8.	No obstacles on the road		
F3—Layout	9.	Overall layout of the ORC	253	0.866
	10.	Rationality of the exits		
	11.	Rationality of the green area		
	12.	Rationality of the road		
	13.	Rationality of the fitness equipment arrangement		
F4—Slip-resistance measures	14.	Slip-resistance measures of in and out the building	257	0.936
	15.	Slip-resistance measures on the road		
	16.	Slip-resistance measures on the steps and ramps		
F5—Noise	17.	Decoration noise	258	0.763
	18.	Transportation noise		
	19.	Domestic noise		
F6—Poor air quality	20.	Life waste odors	257	0.910
	21.	Pungent odor from nearby factory		
	22.	Dust and smog in the ORC		
F7—Lighting	23.	Lighting on the road at night	256	0.938
	24.	Lighting on the public recreational area at night		
	25.	Lighting in the stairway at daytime		
	26.	Lighting in the stairway at night		
F8—Greenery	27.	Green coverage situation in the ORC	205	0.982
	28.	Maintenance of the greenery		
	29.	Beauty of the greenery		
	30.	Quality of greenery maintenance		
F9—Handrail	31.	Handrails along the stairway	244	0.780
	32.	Handrails along the steps outside the building		
	33.	Handrails along the long ramps		
F10—Security	34.	Reliability of the fence around the ORC	206	0.884
	35.	Reliability of building cell gate		
	36.	The real-time monitoring of the ORC		
	37.	Reliability of the security systems		
	38.	Regular check and registration at the ORC gate		
F11—Cleaning	39.	Dustmen clean the road regularly	257	0.882
	40.	The dustman emptied the dustbin every day		
	41.	The dustman cleaned the stairway every day		
F12—Fitness equipment	42.	Convenience of fitness equipment	247	0.955
	43.	Variety of fitness equipment		
	44.	Safety of fitness equipment		
	45.	Maintenance of fitness equipment		
F13—Seating	46.	Quantity of seats	257	0.959
	47.	Quality of seats		
	48.	Location of seats		
F14—Staff	49.	Quantity of staff	254	0.896
	50.	Quality of staff		
	51.	Attitude of staff to seniors		
	52.	Processing efficiency of residents’ concerns		

**Table 4:** Reliability analysis of ODAs factors in ORCs.

Factors	Items	Details	Valid Observations	Alpha (α)
ODAS1—Social activities	1.	Visiting friends in this ORC	241	0.757
	2.	Visiting family members		
	3.	Chatting with neighbors		
ODAS2—Leisure activities	4.	Strolling	243	0.724
	5.	Walking with vigorous strides		
	6.	Doing exercise (tai chi, square dance)		
	7.	Using fitness equipment		
	8.	Playing cards/Mahjong		
ODAS3—Utilitarian-type activities	9.	Going to the post office	255	0.798
	10.	Going to the supermarket		
	11.	Going to the market		
	12.	Going to the bank		
	13.	Going to the hospital		
ODAS4—Nature-exposure activities	14.	Basking	256	0.898
	15.	Enjoying the grass and flowers		
	16.	Having fresh air in the greeneries		

**Table 5:** Correlation between ODAs and factors of ODAs.

Factors	ODA1	ODA2	ODA3	ODA4
	SA	LA	UTA	NEA
ODAS1—Social activities	1.000 **			
ODAS2—Leisure activities	0.477 **	1.000 **		
ODAS3—Outdoor UTA	0.537 **	0.510 **	1.000 **	
ODAS4—Nature-exposure activities	0.616 **	0.409 **	0.218 *	1.000 **
F1—Stairway accessibility	0.189	0.173	0.336 **	0.223 **
F2—Road accessibility	0.232 **	0.256 **	0.157 *	0.230 **
F3—Layout	0.246 **	0.108	0.261 **	0.365 **
F4—Slip-resistance measures	0.245 **	0.272 **	0.064	0.127
F5—Noise	−0.417 **	−0.138	−0.221	−0.422 **
F6—Poor air quality	−0.292 **	0.016	−0.269 **	−0.287 **
F7—Lighting	0.060	−0.070	−0.120	0.160
F8—Greenery	0.241 **	0.071	−0.429 **	0.252 **
F9—Handrail	0.020	−0.163	0.026	0.062
F10—Security	0.006	−0.073	−0.019	−0.023
F11—Cleaning	0.222 **	0.019	−0.317 **	0.244 **
F12—Fitness equipment	0.214	0.092	−0.427 **	0.235 **
F13—Seating	0.185	0.158	−0.404 **	0.236 **
F14—Staff	−0.029	0.293 **	−0.086	−0.009

Note: total sample size = 258; SA—social activities; LA—leisure activities; ODAS—outdoor daily activities; NEA—nature-exposure activities. **—Correlation is significant at the 0.01 level (2-tailed). *—Correlation is significant at the 0.05 level (2-tailed).

**Table 6:** Regression models of ODAs for seniors in ORCs.

	Model	B	Std. Error	t	Sig.	VIF	R	Adjusted R^2^	Significance (ANOVA)
1.	Social activities		←		OE factors				
	(Constant)	2.042	0.214	9.556	0.000		0.373	0.121	0.000
	F4—Slip-resistance measures	0.128	0.058	2.221	0.027	1.069			
	F5—Noise	−2.042	0.214	−9.556	0.000	1.069			
2.	Leisure activities		←		OE factors				
	(Constant)	1.281	0.303	4.229	0.000		0.420	0.159	0.000
	F14—Staff	0.242	0.100	2.411	0.017	1.474			
	F8—Greenery	0.248	0.092	2.706	0.007	1.864			
	F4—Slip-resistance measures	0.313	0.086	3.636	0.000	1.551			
	F2—Road accessibility	0.219	0.089	2.466	0.015	1.443			
3.	Activities of daily living		←		OE factors				
	(Constant)	0.285	0.209	10.751	0.000		0.593	0.327	0.000
	F8—Greenery	−0.298	0.056	−0.293	0.000	1.239			
	F1—Stairway accessibility	0.157	0.060	0.637	0.010	1.109			
	F13—Seating	−0.108	0.041	−0.626	0.010	1.196			
	F4—Slip-resistance measures	0.120	0.052	0.313	0.023	1.264			
4.	Nature-exposure activities		←		OE factors				
	(Constant)	1.629	0.303	5.370	0.000		0.404	0.156	0.000
	F3—Layout	0.398	0.088	4.531	0.000	1.074			
	F6—Poor air quality	−0.216	0.074	−2.635	0.004	1.108			
	F8—Greenery	0.168	0.090	1.865	0.036	1.108			

Notes: missing data processing method: mean substitution; observations = 258; Sig. = significance; VIF = variance inflation factor.

## Data Availability

Data are available on request due to privacy restrictions. The data presented in this study may be available on request from the corresponding author and with the authorization of the funding origination.

## Supplementary Materials

The following are available online at https://www.mdpi.com/article/10.3390/ijerph18147500/s1, File S1: Questionnaire development, File S2: Levene’s Test.
