# Supplementary material for: Investigating the Association between Outdoor Environment and Outdoor Activities for Seniors Living in Old Residential Communities
Source: Int J Environ Res Public Health. 2021 Jul 14;18(14):7500. doi: 10.3390/ijerph18147500 (PMC8307385; doi:10.3390/ijerph18147500)
Supplement: Supplementary file 1 [file ijerph-18-07500-s001.zip › ijerph-1196114-suppl/Supplementary1-Questionnaire development.pdf]

International Journal of
Environmental Research and Public Health

MDPI

Supplementary Material

Investigating the Association between Outdoor Environment and Outdoor Activities for Seniors Living in Old Residential Communities

Shiwang Yu [1], Na Guo [2], Caimiao Zheng [1], Yu Song [3] and Jianli Hao [4,*]

[1] School of Civil Engineering, Sanjiang University, Nanjing 210012, China; Sherwood.s.w.yu@gmail.com (S.Y.); 12017082031@stu.sju.edu.cn (C.Z.)
[2] College of Economic and Management, Nanjing Institute of Industry Technology, Nanjing 210023, China; Guon@niit.edu.cn
[3] XIPU Institution, Xi'an Jiaotong-Liverpool University, Suzhou 215123, China; yu.song@xjtlu.edu.cn
[4] Department of Civil Engineering, Xi'an Jiaotong-Liverpool University, Suzhou 215123, China
* Correspondence: jianli.hao@xjtlu.edu.cn

Questionnaire development

Due to the different types of residential communities, the measurement tools of residential communities in western countries were not firstly considered and the existing Chinese government standards and questionnaires relating to Chinese residential communities were given the priority to measure these factors. Of the fifty-three questions, fifteen questions were adopted from the original ones; twenty-nine questions were adopted after minor adaptation from the original questions; and nine questions were revised to fit the real situation of ORC. Among all the fifty-three questions, most of them were responded with the missing data less than ten and only four questions have more than fifty missing values. All the questions in this study and the original ones as well as the missing data were summarized in Table S1.

Accessibility of the ORCs mainly included four factors in this study. Elevators were not installed in the most of the old residential communities (ORCs) in China [1]. Most of the older adults have to go out through stairway. The measurement of stairway accessibility (F1) includes barrier-free for seniors using wheelchair or walking stick, cleanliness of the stairway, non-slip tiles of the stairway and the ramps set at steps. Of all the four questions, the missing data of Q1 and Q3 were one and five respectively. Road accessibility (F2) was measured by five questions including ease of walking on, no hold pots and uneven surface and so on. Most of the questions were fully responded and the missing data of Q9 was one hundred and eight. Layout (F3) of the ORC was measured by five questions adopted from existing literature (i.e., [2]). Q12 was fully responded; Q10, Q11 and Q13 had one missing data; and Q14 had five missing data. Slip-resistance measures of outdoor environment was measured by three questions adapted from the existing research on indoor environment (i.e., [3]). All the three questions had one missing data.

Physical environment in ORCs were comprised by three factors in this study. Noise (F5) was measured by three questions adopted and adapted from the existing research (i.e., [4]). All the three questions were fully responded. Poor air quality (F6) was measured by three questions which were minorly adapted from the existing research (i.e., [5]). All the questions were fully responded except Q23 which had one missing data. Lighting (F7) was measured by four questions covering the significant areas for the senior adults. All the questions were adapted from the existing research (i.e., [6]). Of the four questions of lighting (F7), Q24, Q26 and Q27 had one missing data; the number of missing data for Q25 was two.

Supporting facilities in ORC included seven factors in this research. Greenery (F8) was measured by four questions which were adopted from the government standard after minor adaptation [7]. Two questions (Q30 and Q31) were fully responded. The number of missing data for Q28 and Q29 were two and fifty-two respectively. The handrail (F9) was measured by three questions adapted from the existing research (i.e., [6]). The number of missing data for Q32, Q33 and Q34 were five, nine and thirteen respectively. Five questions and three questions were adopted from the government standard after minor adaptation to measure security (F10) and cleaning (11). As for security factor (F10), Q35 and Q37 had one missing data; Q36 had two missing data; and Q38 and Q39 had fifty-one missing data. All the questions of cleaning (F11) were fully responded except Q40 which had one missing data. Four questions were adopted from the existing research to measure fitness equipment (i.e., [8]). The number of missing data for Q43 was eleven and the number of missing data for other three questions were both ten. All the three questions to measure seating (F13) were adopted directly from the existing research (i.e., [9]). The number of missing data for all the questions of seating (F13) was one. The staff (F14) factor was measured by four questions which were all adopted from the government standard after minor adaptation. Q53 had four missing data and the other three questions were fully responded.

Table S1. Details of questions in this research and their original ones.

Factors	Items	Questions in the research	Original questions	Reference	Remarks	Missing data
F1-Stairway accessibility	1.	Seniors using wheelchair or walking stick can go through	Indoor barrier-free access	[10]	***	1
	2.	No stuff stacked along the stairway	Cleanliness of public areas	[11]	***	0
	3.	Tiles used in stairway was non-slip	Non-slip finishes provided in common area (e.g., stairs)	[3]	**	5
	4.	Ramps were set at steps	Accessibility at oblique plane	[11]	**	0
F2-Road/path accessibility	5.	Roads/paths are easy to walk on	Bike/pedestrian trails accessible	[12]	**	0
	6.	Manhole covers are kept well	Well-maintained sidewalks	[12]	***	0
	7.	No hollow and holes on the pavement	Pavement cracked, uneven	[13]	**	0
	8.	No obstacles on the road	Pedestrian trails accessible	[12]	**	0
	9.	Split flow of people and vehicles	Sidewalks separated from the roads by parked cars	[12]	**	108
F3-Layout	10.	Overall layout of the ORC	Residential area planning and layout	[2]	*	1
	11.	Rationality of the exits	Rationality of the exits	[2]	*	1
	12.	Rationality of the green area	Rationality of the green area in residential area	[2]	*	0
	13.	Rationality of the road	Rationality of the road in residential area	[2]	*	1
	14.	Rationality of the fitness equipment arrangement	Rationality of the public facilities (e.g., fitness equipment) arrangement	[2]	*	5
F4- Slip-resistance measures	15.	Slip-resistance measures of in and out the building	Indoor barrier-free	[3]	***	1
	16.	Slip-resistance measures on the road	Non-slip finishes provided in the corridors	[3]	***	1
	17.	Slip-resistance measures on the steps and ramps	Non-slip finishes provided in common area (e.g., ramps)	[3]	**	1
F5-Noise	18.	Decoration noise	Renovation noise	[4]	*	0
	19.	Transportation noise	Road noise	[4]	*	0
	20.	Domestic noise	Commercial noise	[4]	**	0
F6-Poor air quality	21.	Life waste odors	Life waste odors due to the untimely disposal	[5]	**	0
	22.	Pungent odor from nearby factory	Pungent odor from nearby factory	[5]	**	0
	23.	Dust and smog in the ORC	Transportation dust and smog	[5]	**	1
F7-Lighting	24.	Lighting on the road at night	artificial light in common areas	[6]	***	1
	25.	Lighting on the public recreational area at night	artificial light in common areas	[6]	***	2
	26.	Lighting in the stairway at daytime	natural light in common areas	[6]	***	1
	27.	Lighting in the stairway at night	artificial light in staircases	[6]	**	1
F8-Greenery	28.	Green coverage situation in the ORC	Many trees, shrubs, grasses and other plants are planted and growing well.	[7]	**	2
	29.	Maintenance of the greenery	All kinds of trees, shrubs, grasses and other plants are regularly watered, fertilized, loosened and sprayed.	[7]	**	52
	30.	Beauty of the greenery	All kinds of trees, shrubs, grasses and other plants are trimmed neatly and **beautifully**.	[7]	**	0
	31.	Quality of greenery maintenance	Make and implement greenery maintenance plan; no damage, trampling, or occupation of greenery space.	[7]	**	0
F9-Handrail	32.	Handrails along the stairway	Handrail of stairs	[6]	*	5
	33.	Handrails along the steps outside the building	Handrail in corridor and lobby	[6]	***	9
	34.	Handrails along the long ramps	Accessibility at oblique plane	[6]	**	13
F10-Security	35.	Reliability of the fence around the ORC	Regular check the **fence**, stairs, windows and building cell gate every day. If any damage is found, it shall be repaired and maintained in time.	[7]	**	1
	36.	Reliability of building cell gate	Regular check the fence, stairs, windows and **building cell gate** every day. If any damage is found, it shall be repaired and maintained in time.	[7]	**	2
	37.	The real-time monitoring of the ORC	The staff in security monitoring room is 24 hours on duty.	[7]	**	1

Factors	Items	Questions in the research	Original questions	Reference	Remarks	Missing data
	38.	Reliability of the security systems	Security systems like monitoring facilities and access control system are running well	[7]	**	51
	39.	Regular check and registration at the ORC gate	Regular check the entry and exit of visitors and vehicles.	[7]	**	51
F11-Cleaning	40.	Dustmen clean the road regularly	Clean up public places and roads in time	[7]	**	1
	41.	The dustman emptied the dustbin every day	No confetti, cigarette butts, plastic bags and other wastes in public places such as roads, green spaces, stairway, and parking lots every day.	[7]	**	0
	42.	The dustman cleaned the stairway every day	No confetti, cigarette butts, plastic bags and other wastes in public places such as roads, green spaces, stairway, and parking lots every day.	[7]	**	0
F12-Fitness equipment	43.	Convenience of Fitness equipment	Convenience of Fitness equipment	[8]	*	11
	44.	Variety of Fitness equipment	Variety of Fitness equipment	[8]	*	10
	45.	Safety of Fitness equipment	Safety of Fitness equipment	[8]	*	10
	46.	Maintenance of Fitness equipment	Maintenance of Fitness equipment	[8]	*	10
F13-Seating	47.	Quantity of seats	Quantity of seats	[9]	*	1
	48.	Quality of seats	Quality of seats	[9]	*	1
	49.	Location of seats	Location of seats	[9]	*	1
F14-Staff	50.	Quantity of staff	Enough staff	[7]	**	0
	51.	Quality of staff	All the staff should hold the professional job certificate.	[7]	**	0
	52.	Attitude of staff to seniors	The attitude of the service staff should be warm and patient, and the manners should be civilized and polite	[7]	**	0
	53.	Processing efficiency of residents' concerns	The timely handling rate of complaints should be 100%.	[7]	**	4

Note: * Original questions; ** Minor changed questions; *** questions which were changed to fit the real situation of the ORC.

Reference

1. Guo, B.; Zhang, L.; Li, Y. Research on the path of residents' willingness to upgrade by installing elevators in old residential quarters based on safety precautions. *Saf. Sci.* **2019**, *118*, 389–396, doi:10.1016/j.ssci.2019.05.038.
2. Sun, Y.X. Study on the planning and layout of commercial residential districts in small towns in Nanxian. Master's Thesis, Hunan Normal University, Changsha, China, 2012.
3. Leung, M.; Yu, J.; Chow, H. Impact of indoor facilities management on the quality of life of the elderly in public housing. *Facilities* **2016**, *34*, 564–579, doi:10.1108/F-06-2015-0044.
4. Ma, J.; Li, C.; Kwan, M.-P.; Chai, Y. A Multilevel Analysis of Perceived Noise Pollution, Geographic Contexts and Mental Health in Beijing. *Int. J. Environ. Res. Public Health* **2018**, *15*, 1479, doi:10.3390/ijerph15071479.
5. Sun, M.X. Research on Influencing Factors and Prediction of Air Quality in Wujiaqu City. Master's Thesis, Shihezi University, Shihezi, China, 2016.
6. Leung, M.; Liang, Q.; Pynoos, J. The effect of facilities management of common areas on the environment domain of quality of life or older people in private buildings. *Facilities* **2019**, *37*, 234–250, doi:10.1108/F-03-2017-0030.
7. Department of Housing and Urban-Rural Development of Jiangsu Province. Evaluation Standards for Service Quality of Provincial Demonstration Facilities Management Projects in Jiangsu Province. Available online: http://jsszfhcxjst.jiangsu.gov.cn/art/2019/1/17/art_49384_8895272.html (accessed on 26 March 2021).
8. Tang, R.P. Analysis of the Problems and Countermeasures in the Safety Management of Public Sports Facilities in Urban Communities. Master's Thesis, Hunan Normal University, Changsha, China, 2018.
9. Tang, S.R. Research on the Current Situation and Countermeasures of the Humanized Design of Outdoor Public Space Environment in the Land-lost Farmers Settlement Community. Master's Thesis, Sichuan Agricultural University, Sichuan, China, 2014.
10. Leung, M.; Famakin, I.; Kwok, T. Relationships between indoor facilities management components and elderly people's quality of life: A study of private domestic buildings. *Habitat Int.* **2017**, *66*, 13–23, doi:10.1016/j.habitatint.2017.05.002.
11. Leung, M.; Famakin, I.O.; Olomolaiye, P. Effect of facilities management components on the quality of life of Chinese elderly in care and attention homes. *Facilities* **2017**, *35*, 270–285, doi:10.1108/F-03-2016-0032.
12. Brownson, R.C.; Chang, J.J.; Eyler, A.A.; Ainsworth, B.E.; Kirtland, K.A.; Saelens, B.E.; Sallis, J.F. Measuring the Environment for Friendliness Toward Physical Activity: A Comparison of the Reliability of 3 Questionnaires. *Am. J. Public Health* **2004**, *94*, 473–483, doi:10.2105/AJPH.94.3.473.
13. Gitlin, L.N.; Mann, W.; Tomit, M.; Marcus, S.M. Factors associated with home environmental problems among community-living older people. Disabil. *Rehabil.* **2001**, *23*, 777–787, doi:10.1080/09638280110062167.
