# Supplementary material for: Investigating the Association between Outdoor Environment and Outdoor Activities for Seniors Living in Old Residential Communities
Source: Int J Environ Res Public Health. 2021 Jul 14;18(14):7500. doi: 10.3390/ijerph18147500 (PMC8307385; doi:10.3390/ijerph18147500)
Supplement: Supplementary file 1 [file ijerph-18-07500-s001.zip › ijerph-1196114-suppl/supplementary2-Levene's test.pdf]

International Journal of
Environmental Research and Public Health

MDPI

Supplementary Material

Investigating the Association between Outdoor Environment and Outdoor Activities for Seniors Living in Old Residential Communities

Shiwang Yu [1], Na Guo [2], Caimiao Zheng [1], Yu Song [3] and Jianli Hao [4,*]

[1] School of Civil Engineering, Sanjiang University, Nanjing 210012, China; Sherwood.s.w.yu@gmail.com (S.Y.); 12017082031@stu.sju.edu.cn (C.Z.)
[2] College of Economic and Management, Nanjing Institute of Industry Technology, Nanjing 210023, China; Guon@niit.edu.cn
[3] XIPU Institution, Xi'an Jiaotong-Liverpool University, Suzhou 215123, China; yu.song@xjtlu.edu.cn
[4] Department of Civil Engineering, Xi'an Jiaotong-Liverpool University, Suzhou 215123, China
* Correspondence: jianli.hao@xjtlu.edu.cn

Levene test of ODAs

Levene's test was conducted to check the differences in variables for the respondents in fourteen surveyed ORCs. The p-value was set as 0.0007 to control Type I error. The results were summarised in Table S1 and S2. All the P values of ODAs were larger than 0.001, which means this research did not violate the homogeneity of variances assumption (refer to Table S1). The variable data of fourteen ORCs were finally combined in this study.

Table S1. The details of means, ANOVA and Levene test for four types of outdoor daily activities.

Factors	Mean	S.D.	ANOVA		Homogeneity tests	
			F.	Sig.	Levene statistic	Sig.
ODAS1-Social activities	3.5104	0.79057	0.833	0.640	1.604	0.073
ODAS2-Leisure activities	2.7119	0.95812	2.294	0.005	1.133	0.328
ODAS3- Outdoor UTA	2.6808	0.90783	1.006	0.450	2.256	0.006
ODAS4- Nature exposure activities	3.6237	1.01379	0.679	0.805	2.360	0.004

Note: The questionnaire comprised 69 questions, and the adjusted p-value was 0.0007 (0.05/69) to control Type I error.

Table S2. Comparison of means of four types of ODAs among fourteen surveyed ORCs.

Factors	ORCs	N	Mean	Standard Deviation	Standard Error
ODAS1-Social activities	1.00	16	3.7083	0.7876	0.1969
	2.00	14	3.7381	1.1996	0.3206
	3.00	17	3.1569	0.7371	0.1788
	4.00	18	3.6111	1.1217	0.2644
	5.00	16	3.7708	1.0163	0.2541
	6.00	16	3.6042	1.1037	0.2759
	7.00	15	3.2667	1.1423	0.2949
	8.00	18	3.4074	1.2862	0.3032
	9.00	16	3.8125	0.6321	0.1580
	10.00	16	3.6875	0.9307	0.2327
	11.00	14	3.7619	0.9734	0.2602
	12.00	17	3.8824	1.0337	0.2507
	13.00	16	3.7083	1.1345	0.2836
	14.00	16	3.3542	0.9386	0.2347
	15.00	14	3.5238	1.2175	0.3254
	16.00	17	4.0000	0.8165	0.1980
	Total	256	3.6237	1.0138	0.0634
ODAS2-Leisure activities	1.00	14	3.6429	0.7334	0.1960
	2.00	10	4.0667	0.8721	0.2758
	3.00	17	3.2745	0.6896	0.1672
	4.00	18	3.4815	0.7857	0.1852
	5.00	16	3.4167	0.8650	0.2162
	6.00	14	3.4524	0.6744	0.1802
	7.00	15	3.3778	0.7854	0.2028

Factors	ORCs	N	Mean	Standard Deviation	Standard Error
	8.00	13	2.8974	0.9849	0.2732
	9.00	16	3.1667	0.4037	0.1009
	10.00	16	3.2917	0.5821	0.1455
	11.00	14	3.9286	0.4563	0.1219
	12.00	17	3.9804	1.1085	0.2688
	13.00	15	3.4000	0.9361	0.2417
	14.00	16	3.5208	0.5301	0.1325
	15.00	13	3.6923	0.6156	0.1707
	16.00	17	3.7255	0.7838	0.1901
	Total	241	3.5104	0.7906	0.0509
ODAS3- Outdoor UTA	1.00	16	2.4500	0.6429	0.1607
	2.00	14	2.3429	1.2586	0.3364
	3.00	17	2.4000	0.8185	0.1985
	4.00	18	2.9000	1.1381	0.2683
	5.00	15	2.8133	1.1526	0.2976
	6.00	15	2.6533	0.8798	0.2272
	7.00	15	2.6933	0.8345	0.2155
	8.00	10	2.3400	0.8003	0.2531
	9.00	16	2.7500	0.3967	0.0992
	10.00	16	2.8125	1.0942	0.2735
	11.00	14	2.6571	0.9095	0.2431
	12.00	17	3.0706	1.2287	0.2980
	13.00	17	3.0118	1.0618	0.2575
	14.00	15	2.8400	0.8559	0.2210
	15.00	14	2.4000	0.8376	0.2239
	16.00	14	3.0286	0.9277	0.2479
	Total	243	2.7119	0.9581	0.0615
ODAS4- Nature exposure activities	1.00	16	2.7125	0.4674	0.1169
	2.00	14	2.5857	1.1883	0.3176
	3.00	17	2.4941	0.9114	0.2210
	4.00	18	2.3778	1.1780	0.2777
	5.00	16	2.8125	0.7173	0.1793
	6.00	15	2.9333	0.9933	0.2565
	7.00	15	2.5600	0.6895	0.1780
	8.00	18	2.3889	0.9393	0.2214
	9.00	16	2.5250	0.5508	0.1377
	10.00	16	2.8250	0.8418	0.2105
	11.00	14	2.7571	0.8985	0.2401
	12.00	17	3.0000	1.2865	0.3120
	13.00	17	2.7647	1.1073	0.2686
	14.00	15	2.8667	0.9730	0.2512
	15.00	14	2.6714	0.7710	0.2061
	16.00	17	2.6941	0.6290	0.1525
	Total	255	2.6808	0.9078	0.0569
